# Interprofessional Collaboration between Residents and Nurses in General Internal Medicine: A Qualitative Study on Behaviours Enhancing Teamwork Quality

**DOI:** 10.1371/journal.pone.0096160

**Published:** 2014-04-25

**Authors:** Virginie Muller-Juge, Stéphane Cullati, Katherine S. Blondon, Patricia Hudelson, Fabienne Maître, Nu V. Vu, Georges L. Savoldelli, Mathieu R. Nendaz

**Affiliations:** 1 Unit of Development and Research in Medical Education (UDREM), Faculty of Medicine, University of Geneva, Geneva, Switzerland; 2 Quality of Care Service, University Hospitals of Geneva, Geneva, Switzerland; 3 Institute of Demographic and Life Course Studies, Faculty of Economic and Social Sciences, University of Geneva, Geneva, Switzerland; 4 Division of General Internal Medicine, University Hospitals of Geneva, Geneva, Switzerland; 5 Department of Community Medicine, Primary Care and Emergency Medicine, University Hospitals of Geneva, Geneva, Switzerland; 6 Division of Anaesthesiology, University Hospitals of Geneva, Geneva, Switzerland; University of Stirling, United Kingdom

## Abstract

**Background:**

Effective teamwork is necessary for optimal patient care. There is insufficient understanding of interactions between physicians and nurses on internal medicine wards.

**Objective:**

To describe resident physicians’ and nurses’ actual behaviours contributing to teamwork quality in the setting of a simulated internal medicine ward.

**Methods:**

A volunteer sample of 14 pairs of residents and nurses in internal medicine was asked to manage one non-urgent and one urgent clinical case in a simulated ward, using a high-fidelity manikin. After the simulation, participants attended a stimulated-recall session during which they viewed the videotape of the simulation and explained their actions and perceptions. All simulations were transcribed, coded, and analyzed, using a qualitative method (template analysis). Quality of teamwork was assessed, based on patient management efficiency and presence of shared management goals and of team spirit.

**Results:**

Most resident-nurse pairs tended to interact in a traditional way, with residents taking the leadership and nurses executing medical prescriptions and assuming their own specific role. They also demonstrated different types of interactions involving shared responsibilities and decision making, constructive suggestions, active communication and listening, and manifestations of positive team building. The presence of a leader in the pair or a truly shared leadership between resident and nurse contributed to teamwork quality only if both members of the pair demonstrated sufficient autonomy. In case of a lack of autonomy of one member, the other member could compensate for it, if his/her own autonomy was sufficiently strong and if there were demonstrations of mutual listening, information sharing, and positive team building.

**Conclusions:**

Although they often relied on traditional types of interaction, residents and nurses also demonstrated readiness for increased sharing of responsibilities. Interprofessional education should insist on better redefinition of respective roles and reinforce behaviours shown to enhance teamwork quality.

## Introduction

This paper reports on the second phase of a project aimed at exploring the perceptions of resident physicians and nurses about their roles [1] and observing their actual behaviours in practice. Effective teamwork is necessary for optimal patient care and is associated with better patient outcome [2], [3], [4], [5]. The hospital is a context of complex clinical practice, heavy workload, and numerous team shifts. The quality of interprofessional and multidisciplinary collaboration is crucial and has been shown to influence patients’ readmission to intensive care unit [6], patients’ length of stay [7], and other outcomes [8], [9], [10], [11]. Effective teamwork requires specific cognitive, technical, and affective competence, as determined in a focus-group study conducted in the field of primary care [12]. Five general characteristics of team effectiveness emerged from this study: understanding and respecting team members’ roles; recognizing that creating and maintaining teamwork is an ongoing process; sharing a common understanding of primary health care; having the practical “know-how” for sharing patient care; and communication [12]. Communication was identified as the essential factor in effective teams [9], [12], [13]: “Improving communication would increase understanding, co-operation, and collaboration among team members” [12]. In another focus-group study [14], clear goals and attention to teamwork were identified as factors needed for team effectiveness in primary care. In a review paper, Leonard [15] defined the following components of effective teamwork and communication: structured communication, effective assertion/critical language, mutual respect and appreciation (“psychological safety”), situational awareness, and effective leadership.

Most studies in the field of interprofessional collaboration have been conducted in intensive-care or reanimation settings. Their findings may not strictly apply to internal medicine contexts, in which ill-defined problems, due to increased comorbidities and aging of patients, complicate patient management and require a high-level interprofessional collaboration [16]. Thus, there is a need to improve our understanding of interactions between physicians and nurses on internal medicine wards.

In a first step of our project [1], we conducted individual interviews with resident physicians and nurses from the Division of General Internal Medicine to explore perceptions and expectations of their professional roles, for their own and the other profession. Additionally, participants filled out a questionnaire asking their own intended actions as well as their expected actions from the other professional in response to 11 clinical scenarios. We found a lack of shared perceptions and expectations between the physicians and the nurses regarding nurses’ autonomy in patient management, their participation in the decision-making process, professional interdependence, and physicians’ implication in teamwork. We also showed that nurses’ intended actions differed from physicians’ expectations mainly regarding nurses’ autonomy in patient management: nurses considered themselves more autonomous in the initial patient management than physicians thought. As a second step, the present study aimed at describing physicians’ and nurses’ actual characteristics and behaviours that contribute to teamwork quality in the setting of a simulated hospital internal medicine ward.

## Methods

The overall project was approved by the research ethics committee of the University Hospitals of Geneva. A complete review was waived by this committee. Participants received a written description of the project and gave written consent for participation and for the use of the audio- and video-recorded material. The participants were guaranteed that their anonymity would be preserved and that the data would not be used for assessment purposes.

### Setting and Participants

A total of 33 resident physicians and 54 nurses in the Division of General Internal Medicine at the University Hospitals of Geneva, Switzerland were eligible at the time of the study. Resident physicians (“residents” or house officers in the UK) are physicians in graduate training leading to a medical specialty. They usually have one to five years of graduate experience. The proportion of males among the participants was respectively 45% and 30%. The project was presented in regular physician and nurse staff meetings for recruitment. Participants volunteered for the study and were included if residents had one to five years of experience in the internal medicine residency program and if nurses were actively working on the internal medicine ward at the time of the study. Overall, we recruited 14 pairs of residents and nurses.

### Data Collection

After having been interviewed about their perceptions and expectations regarding their own and the other profession [1], each nurse was randomly teamed up with a resident and each pair was asked to manage one non-urgent (Case 1) and one urgent (Case 2) clinical case in a simulated internal medicine ward. These simulations took place at the simulation centre of our hospital, using a high-fidelity manikin able to reproduce several clinical situations (SIMULHUG, http://simulationmedicale.hug-ge.ch/). The sequence of the two simulations (Case 1 presented first) was the same for all pairs because Case 1 allowed participants to familiarize with the simulation setting while Case 2, a more challenging situation, might better uncover strengths and weaknesses of the interactions. For four clinical complaints commonly encountered on an internal medicine ward we developed one non-urgent and one urgent case scenario that require an interprofessional management. The details of each case are displayed in Table 1.

**Table 1:** Details of each case with their relative relevant diagnostic hypotheses*.

Complaint	Non-urgent clinical case (Case 1)	Urgent clinical case (Case 2)
Dyspnoea	Exacerbation of severe COPD	Cardiac failure due to rapid atrial fibrillation in a COPD patient. Pulmonary embolism to rule out.
Melena	Upper gastro-intestinal haemorrhage without hemodynamic instability	Upper gastro-intestinal haemorrhage in an anticoagulated patient, with hemodynamic instability
Fever	Endocarditis due to infected peripherical catheter	Endocarditis due to infected peripherical catheter with sepsis and oliguria
Epigastralgia	Gastric ulcer	Inferior NSTEMI

* COPD: chronic obstructive pulmonary disease; NSTEMI: non-ST elevation myocardial infarction.

After each simulation, participants attended an individual stimulated-recall session [17] with one of the investigators, during which they viewed the videotape of the simulation and explained their actions and perceptions.

### Analysis

All simulations were audio and video-recorded, transcribed verbatim, coded, and analyzed qualitatively using the template analysis approach [18], [19], [20]. In this type of analysis, researchers develop a “template” of *a priori* codes that represent themes expected to be relevant to the analysis, based on experience, review of relevant literature, or initial review of the data set. Codes are usually presented in a hierarchical manner, with broad themes encompassing successively narrower, more specific ones. However, these *a priori* codes may be modified, rearranged or eliminated, depending on their utility and appropriateness to the data.

Stimulated recalls were also audio-recorded and transcribed. Their purpose was to understand the events observed during the simulations and resolve any uncertainties while coding.

During the initial interviews with the participants, we identified behaviours important to observe during the simulations. They constituted the basis of our codebook, in addition to selected items from published scales [21], [22], [23], [24]. The codebook was adjusted as needed during the process of coding. The codebook included 30 codes, addressing the following three main themes: a) autonomy and problem analysis, b) technical communication, such as medical prescriptions and their verification, confirmation of executed medical orders (closed loop), call-out of measurement or test results, and c) manifestations of team building, such as providing feedback, helping each other, or expressing positive or negative emotions (Table S1).

A team of three researchers (trio) consisting of an educationalist (VMJ), a physician (GS or MN), and alternately one of the following: a nurse (FM), an anthropologist (PH), a sociologist (SC), or a medical education specialist (NV) first coded the videos independently, using Atlas.ti (ATLAS.ti Scientific Software Development GmbH, Version 7.0.71). Afterwards, they compared and discussed any coding differences until consensus was reached.

After coding each of the simulations, the same trio of researchers provided their overall impression of team members’ characteristics and of quality of teamwork. Quality of teamwork, as defined above by Sargeant and Leonard [12], [15] was evaluated based on researchers’ perceptions of patient management efficiency, shared management goals, and team spirit (Table 2). For each dimension, the researchers provided their overall assessment regarding presence/absence of the following characteristics: autonomy (*i.e.* the ability to initiate and pursue relevant reasoning or actions regarding patient management), positive working atmosphere, mutual listening, traditional role-sharing, and effective teamwork (Table 2). Disagreements among the trio of coders were resolved by discussion until consensus was reached about the absence (0), the partial presence (1), or the strong presence (2) of these dimensions. To facilitate the illustration of these results by radar figures, the global impressions were labelled as a number, which should not be interpreted as a true rating scale.

**Table 2:** Statements allowing researchers to provide their overall impressions on characteristics of the team members and on the quality of patient management.

**Statements** *
The roles are traditional (the resident prescribes, the nurse executes)
The resident-nurse pair works harmoniously during their interactions (absence of conflict, of aggressiveness, etc.)
The team is efficient in patient management
The resident-nurse pair has common goals on patient management
The resident assumes leadership of the patient management
The nurse assumes leadership of the patient management
The nurse listens attentively to resident
The resident listens attentively to nurse
The resident and nurse demonstrate a shared reasoning and shared decision making
The resident demonstrates autonomy in patient management
The nurse demonstrates autonomy in patient management

* For each statement, the researchers gave their global impression about the absence, the partial presence, or the strong presence of each dimension and could additionally provide free comments.

## Results

### Participant Characteristics

Residents were mostly men (male to female ratio 10∶4), whereas nurses were predominantly women (male to female ratio 4∶10). Their mean age was 34 years (residents 31 years, nurses 37 years). Postgraduate mean experience was respectively 4 and 10 years for residents and nurses but all had been in the Division of General Internal Medicine for a similar length of time (residents 3 years, nurses 4 years). The proportion of male pairs was 28.5%, female pairs 28.5%, and mixed pairs 43.0%. Residents had more postgraduate experience in 21% of the pairs, nurses had more postgraduate experience than residents in 71% of pairs, and experience was similar between residents and nurses in 8% of the pairs.

Each of the 28 simulations lasted on average 18 minutes (SD = 1.5, range 15–21). The mean duration of the 56 stimulated-recall sessions was 46 minutes (SD = 6.3, range 28–74).

### Overall Characteristics of Pair Interactions

The evaluation of the different dimensions observed by the coders was plotted on radar charts for each case as a visual support to illustrate the results of our qualitative analysis.

**Figure 1:**
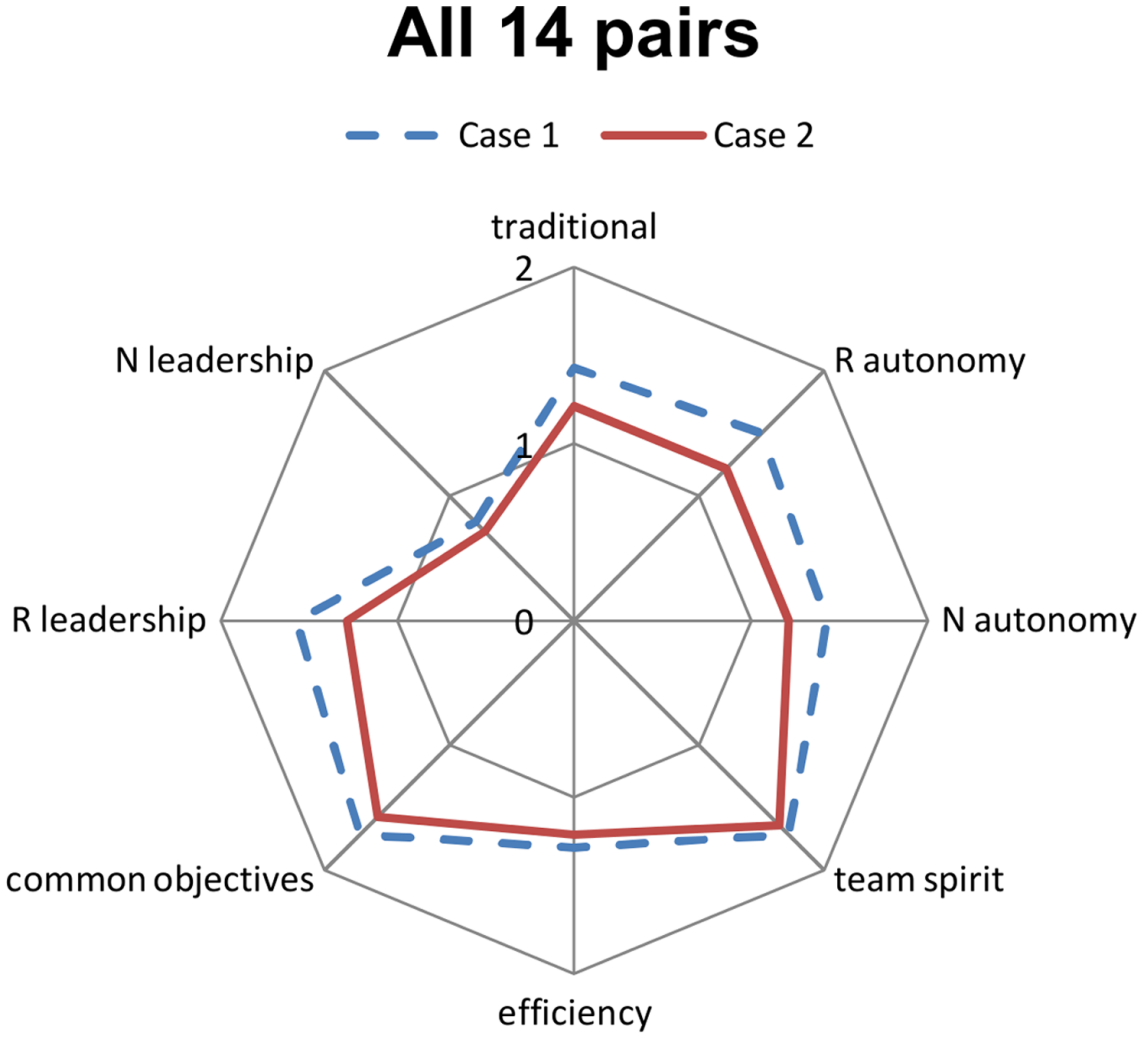
Overall characteristics of the interactions within resident-nurse pairs. The pair functioning was generally considered rather traditional, with the residents taking the leadership more often than nurses and with the nurses executing medical prescriptions and assuming their own specific role regarding patient supervision and care. The pairs were globally autonomous, especially in non-urgent cases, there was a good team spirit, and the team members had common management objectives and managed the patients with good, although not always maximal, efficiency. Each line represents a different case: in blue the non-urgent case (Case 1) and in red the urgent case (Case 2). R: resident; N: nurse. 0: absence of the characteristic; 1: partial presence of the characteristic; 2: strong presence of the characteristic, as determined by consensus among the coders.

Figure 1 represents the overall characteristics of the pair interactions. The pair functioning was generally considered rather traditional, with the residents taking the leadership more often than nurses and with the nurses executing medical prescriptions and assuming their own specific role regarding patient supervision and care [25]. Additionally, team members were generally autonomous, especially in non-urgent cases, there was a good team spirit, and the team members had common management objectives and managed the patients with good, although not always maximal, efficiency.

### Determinants of Teamwork Quality

**Figure 2:**
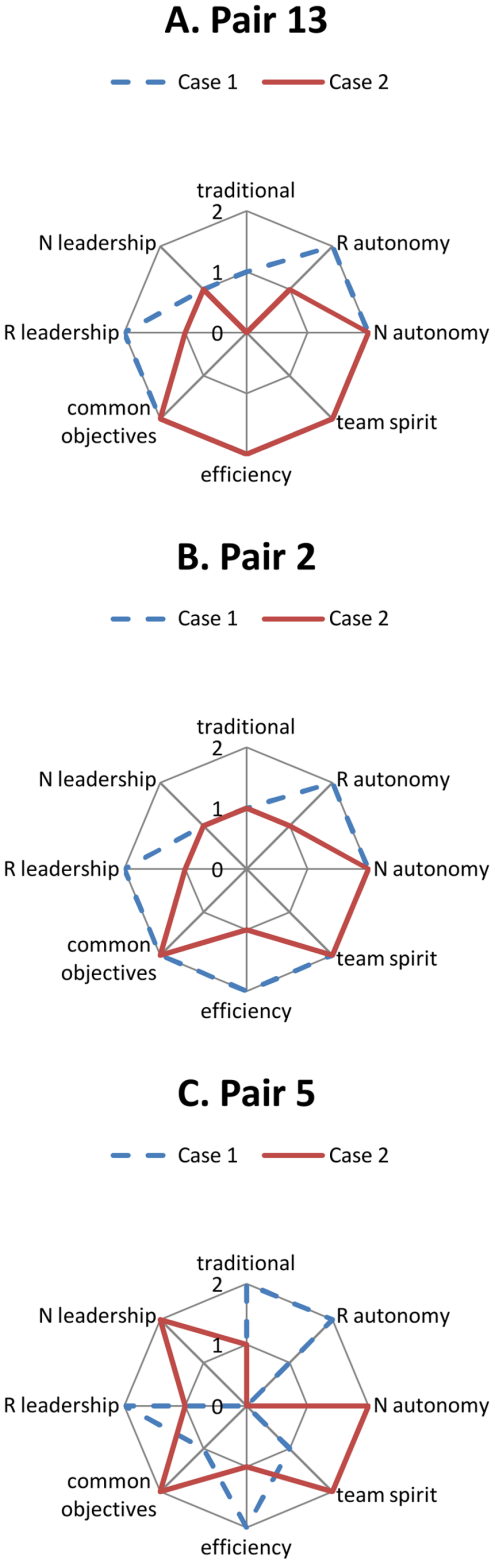
Characteristics of selected resident-nurse pair interactions. A. Pair 13. The presence of a leader in the team (Pair 13, Case 1) or of a truly shared leadership between the resident and the nurse (Pair 13, Case 2) was the first condition for teamwork quality across pairs. B. Pair 2. Leadership was a necessary but insufficient condition. It contributed to teamwork quality only if the leader or both members of the pair demonstrated sufficient autonomy (Pair 2, Case 1). C. Pair 5. Nurse leadership could vary within the same pair, depending on the case type. For example in Pair 5, the resident took the leadership in Case 1 and the nurse tended to stand back, but in the more urgent Case 2 the nurse took the leadership once it appeared that the resident had difficulty in doing so. Each line represents a different case: in blue the non-urgent case (Case 1) and in red the urgent case (Case 2). R: resident; N: nurse. 0: absence of the characteristic; 1: partial presence of the characteristic; 2: strong presence of the characteristic, as determined by consensus among the coders.

The presence of a leader in the team (*e.g.* Figure 2A, Pair 13, Case 1) or of a truly shared leadership between the resident and the nurse (Figure 2A, Pair 13, Case 2) was the first condition for teamwork quality across pairs. Truly shared leadership was present when each member alternatively took a strong leadership for case management or when decisions were really made consensually. This pattern, however, occurred infrequently among the pairs.

Leadership was a necessary but insufficient condition in several cases (*e.g.* Pair 3, Case 1; Pair 4, Case 1; Pair 10, Case 1; Pair 10, Case 2). It contributed to teamwork quality only if the leader or both members of the pair demonstrated sufficient autonomy (*e.g.* Figure 2B, Pair 2, Case 1). If the non-leader member of the group did not demonstrate full autonomy (*e.g.* Figure 2C, Pair 5, Case 1), alternative conditions could nevertheless contribute to teamwork quality:

a) When the nurse understood the situation, was interested in its follow-up, helped the resident understand the case, and communicated on technical aspects (verified the prescriptions and confirmed aloud their execution);

After the resident had listened to the patient’s lungs, the nurse asked: “What did you find at auscultation?” (Pair 5 Case 2).

Nurse to resident, while looking at monitoring: “Do you want me to get the electrocardiography, because you see, the patient has bradycardia” (Pair 4 Case 2).

Nurse to resident, for a bleeding patient: “Yes he is on warfarin but you see, he has also received aspirin” (Pair 14 Case 2).

b) When the team members displayed behaviours enhancing team spirit (such as providing feedback and mutual listening);

Nurse: “… and I also checked blood sugar.” Resident: “Good idea, you did a good job.” (Pair 13 Case 1).

Resident: “Did you give morphine?” Nurse: “Yes I already did it” Resident: “Great, how efficient you are!” (Pair 8 Case 1).

Resident: “I will write down my oral orders to you.” Nurse: “Good, I thank you very much.” (Pair 9 Case 1).

Nurse, while resident tries to sit up the patient: “Do you need any help?” (Pair 6 Case 1).

c) When both members provided explanations and justifications about their decisions.

Resident to nurse, about oxygen debit: “…because you see, I wonder if he has COPD” (Pair 6 Case 1).

Nurse to resident: “…the patient had low blood pressure, tachycardia and saturation was at 84%. I put it on slow debit of oxygen, 2 litres/minute, because he has emphysema.” (Pair 11 Case 2).

These results were confirmed by the analysis of the less efficient situations, occurring exclusively when the pairs managed the more urgent cases (e.g. Figure 2B, Pair 2, Case 2). In these cases, either a leader was missing or when leadership was present, the leader or both members of the pair lacked autonomy. These less efficient pairs also displayed poor mutual listening and weak team spirit. The interactions revealed a tense atmosphere, the absence of positive team building and of technical communication, and the resident’s inability to compensate for the nurse’s lack of autonomy.

### Expressions of Leadership and Autonomy

In previous interviews made with residents and nurses [1], nurses’ leadership and autonomy represented a topic with discordant views: nurses considered themselves more autonomous than residents thought. Therefore, we specifically focused and analyzed these two aspects in the present data. We found that nurses rarely assumed leadership in the clinical situations (in 3 of the 28 interactions, *e.g.* Figure 2C, Pair 5, Case 2). It appeared that the nurse led the clinical management when the resident did not, and when a) the nurse demonstrated enough autonomy for the case management and b) there were manifestations of positive team building, such as mutual listening and feedback, and efficient communication about medical information. Examples of nurses and residents’ manifestations of leadership and autonomy are listed and illustrated in Table 3. Nurse leadership could vary within the same pair, depending on the case type. For example in Pair 5 (Figure 2C), the resident took the leadership in Case 1 and the nurse tended to stand back, but in the more urgent Case 2 the nurse took the leadership once it appeared that the resident had difficulty in doing so.

**Table 3:** Residents’ and nurses’ expressions of leadership and autonomy.

Expressions of leadership and autonomy	Examples
**Residents**	
Request for nurses’ opinion and involve them in patient management	(while on the phone with nurse) *«Are you concerned about the patient? Do you want me to come before the medical round or immediately?» (Resident 6 Case 1)*
	*«No other problem to mention on your side?» (Resident 11 Case 1)*
Give clear medical orders	*«Please could you give 10 mg vitamin K i.v.,* * *stat?» (Resident 7 Case 1)*
Call out results of exams	(after lung auscultation) *«I hear rhonchi on the left side.» (Resident 7 Case 1)*
	(while reading the electrocardiogram) *«He’s got negative T waves on V5 and V6.» (Resident 12 Case 2)*
Plan the sequence of actions	*«We perfuse volume, we administer antibiotics, I’ll call the chief resident and then draw for blood gases.» (Resident 6 Case 1)*
Verify that medical orders are in progress	*«Did you administer omeprazole? Did you give aspirin?» (Resident 9 Case 2)*
Listen attentively to nurses	Observation by coders
**Nurses**	
Collect information from the patient (history and clinical exam)	(speaking to the patient before the resident is paged) *«First let’s check the blood pressure to see if it can explain the dizziness. Do you remember the glucose level from this morning? (Nurse 3 Case 2)*
Demonstrate comprehension of the situation and interest in its follow-up	(observation by the coders) The nurse looks at the blood gases results with the resident (Nurse 5 Case 2) or at the electrocardiogram (Nurse 8 Case 1)
Suggest and give their opinions	*«A pitting edema of the left leg? Perhaps a pulmonary embolism, don’t you think?» (Nurse 12 Case 2)*
Call out results of exams	*«I have a pulse of 44 and 88% saturation.» (Nurse 9 Case 2)*
Verify the medical prescriptions	*«You said 500 ml of saline, right?» (Nurse 4 Case 1)*
Ask for a prescription or a precision on a given prescription	*«How much saline do you want? At what rate?» (Nurse 9 Case 2)*
Help the residents	(observation by the coders) The nurse opens the patient’s gown to help the resident auscultate the thorax. The nurse helps the resident lower or raise the patient’s bed (Nurse 5 Case 2, Nurse 10 Case 1, Nurse 13 Case 1)
Listen attentively to residents	Observation by the coders

* i.v.: intravenous(ly).

## Discussion

The overall functioning of the resident-nurse pairs was rather traditional in our study: The residents took often the leadership of the patient management, made prescriptions and decisions and the nurses executed the medical prescriptions and assumed their own specific roles in patient care and supervision. Concurrently, the first condition contributing to teamwork quality was the presence of a leader in the pair or a truly shared leadership between the resident and the nurse. However, the presence of a leadership was a necessary but insufficient condition as it contributed to teamwork quality only if the leader or both members of the pair demonstrated sufficient autonomy. In case of a lack of autonomy of one member of the pair, the other member could only compensate for it if his/her own autonomy was sufficient and if there was a climate of mutual listening, information sharing, and demonstrations of positive team building.

One of the strengths of our study was to involve residents and nurses working in a context of internal medicine, which has not been widely studied until now. It used simulation technology and offered the possibility to make conclusions based on observations, and not only on self-reports as has been done in the majority of previous studies. Moreover, the data were reviewed and analyzed by a multidisciplinary team of investigators including nurse, physicians, sociologist, anthropologist, and educators, which enriched the interpretation of the observed interactions. This study took place in only one institution and may not be representative of other settings, especially regarding cultural aspects, such as norms and values related to resident-nurse relationships and interactions. Moreover, the participants were young physicians still in training interacting with more experienced nurses, preventing the generalization of our results to any interaction between a physician and a nurse. However, we chose this sampling because it represents the actual setting of most internal medicine departments, in which a clinical situation is usually evaluated by nurses and residents before a more experienced, senior staff member is called. Participants volunteered for the study and were thus potentially biased towards the topic of interprofessional collaboration, which may have influenced their behaviours during the simulations. However, the wide range of behaviours we observed during the simulations makes an important selection bias less likely. Finally, a larger proportion of male residents volunteered for this study within our eligible resident population, which may limit the representativeness of the residents’ behaviours. Male:female ratio of our volunteer nurses was, nevertheless, similar to the ratio in the eligible nurse population. The quality of teamwork was based on subjective perceptions of the researchers. However, each individual perception was confronted to the one of each member of a trio of coders and when disagreement occurred, consensus was reached by returning to the original transcripts of the simulation and of the stimulated recall, thus contributing to judgment reliability.

Our findings support Leonard’s view of effective teamwork in health care [15], in which the following components are essential: Effective leadership, situational awareness, structured communication, effective assertion/critical language, mutual respect and appreciation (“psychological safety”). However, most of the resident-nurse interactions we observed in our study do not completely meet Baggs’ definition of interprofessional collaboration regarding responsibilities, defined as: “sharing responsibilities for solving problems, and making decisions to formulate and carry out plans for patient care” [6]. Responsibility was generally taken by the residents without a true sharing with the nurses, which was at odds with their stated views expressed during previous interviews about their role perceptions, during which they generally valued shared responsibilities [1]. This finding must be interpreted in the light of the historical evolution of both professions and of their interactions, at least in Western Europe [25], [26], [27], [28]. Before early 20^th^ century, nursing was assumed by nuns or volunteers who were living in hospices and were dedicated to patient care and home maintenance [26], under the order of physicians. During the first decades of the 20^th^ century, nursing schools developed, allowing nursing to become a recognized profession. However, the relationship between physicians and nurses remained rooted in traditional roles in which the physician is the sole decision maker and team leader while the nurse was expected to display “discipline, self-control, and obedience”, “wary of independent decision making” [27]. Although this “doctor-nurse game” became less prevalent after the end of the ‘60s, it may still influence the representation of roles and willingness to change [29], [30]. Medical and nursing education may reinforce this representation as long as each profession provides separate training in a strict “silo” manner, and as long as new models for interdisciplinary interactions have not been recognized and implemented [31]. In addition, the site of training and the local culture [32] may represent other modulators of role representations, as well as role models encountered by the trainees, who might also convey, consciously or not, their own concept of physician-nurse interactions. Legal aspects have also to be considered when interpreting our observations. According to Swiss law, nurses are not allowed to diagnose or treat patients without the prescriptions of a physician. Therefore, this may also have contributed to the traditional aspects of the interactions we observed in our study.

Most resident-nurse interactions occurred in a serene atmosphere, with positive displays of teambuilding and mutual listening, with nurses making suggestions and verifying prescriptions. One resident-nurse pair demonstrated a high degree of shared leadership and in other pairs, nurses took the leadership when the resident did not, particularly in more urgent cases at higher stakes for the patient. These observations, as well as the results of previous interviews made with the same participants on their respective role perceptions [1], show that the present generation of residents and nurses may be open to new models of interdisciplinary interactions. This argues for reinforcement of interprofessional education at the pre- and postgraduate levels, as exemplified by existing programs [33], [34], [35], [36], [37]. However, if such programs aspire to success, they must take into account representations of interprofessional interactions in the context of local culture and go beyond the activities limited to medical or nursing schools. They must use clinical teaching materials requiring a high level of relevant interprofessional collaboration. These programs should continue during postgraduate and continuous education and involve all stakeholders concerned by patient care and the organization of the health structures because efforts made during pre-graduate training may be counteracted by resistance to change from those already in the workplace. Leadership is essential, although not sufficient to enhance teamwork quality. On the other hand, too strong a leadership may be perceived as an obstacle to facilitate the sharing of responsibilities. In order for these characteristics to coexist, it seems first important, beyond traditional and historical views, to make the repartition of roles explicit within a resident-nurse pair taking care of a specific patient situation, depending on each one’s experience and specific professional training. Second, even when one member of the pair takes the lead, the role of the other member should be to help, suggest, and verify decisions. Situational leadership and supportive collaboration are enhanced if the working environment is favourable, if there is good communication about the encountered situation, and if there is mutual listening and positive displays of team-building [38]. These ingredients should be part of any interprofessional education and should complement the technical and content training as necessary competencies to be practiced and acquired during interprofessional team training. In this regard our findings are similar to the “non-technical skills” training described in reanimation settings [21], [39], but with specificities related to the problems of internal medicine wards.

Our study suggests a number of areas for future research. It is unclear to what extent participants’ self-reported perceptions about their roles fit their actual performance during the simulations and how much the training setting of the participants might have influenced their perceptions and behaviours. Additionally, it would be worth assessing how the individual reasoning of each member of the team may evolve towards a concept of “team reasoning” and how interprofessional collaboration may influence individual and collective reasoning and decisions of team members. Finally, the impact of an interprofessional education implementing the dimensions related to enhanced teamwork quality raised in this study represents a further area of research.

## Conclusion

Our study found that resident-nurse pairs generally worked together according to traditional professional roles: residents took the lead while nurses executed prescriptions and assumed traditional nursing roles of supervision and care. However, residents and nurses also demonstrated openness to other types of interprofessional interactions, including the sharing of responsibilities and decision-making, mutual listening, exchange of suggestions, and positive displays of team building. Our results suggest a number of dimensions that enhance teamwork quality and that should be addressed in interprofessional education in an internal medicine setting, both at the pre- and postgraduate levels.

## Supporting Information

Table S1 **Main codes used to assess the encounters of nurse-resident pairs.**
(DOCX)
