# Supplementary material for: Interprofessional Collaboration between Residents and Nurses in General Internal Medicine: A Qualitative Study on Behaviours Enhancing Teamwork Quality
Source: PLoS One. 2014 Apr 25;9(4):e96160. doi: 10.1371/journal.pone.0096160 (PMC4000227; doi:10.1371/journal.pone.0096160)
Supplement: Table S1 — Main codes used to assess the encounters of nurse-resident pairs. (DOCX) [file pone.0096160.s001.docx]

**Table S1: Main codes used to assess the encounters of nurse-resident pairs**

| **Theme 1:** autonomy and reflection |
| --- |
| Nurse anticipates resident’s needs in patient care, demonstrates decision making, initiatives, reflection (history taking and physical exam) |
| Nurse anticipates resident’s needs in patient care, demonstrates decision making, initiatives, reflection (tests) |
| Nurse anticipates resident’s needs in patient care, demonstrates decision making, initiatives, reflection (treatment) |
| Nurse makes suggestions and gives his/her opinion |
| Nurse demonstrates comprehension of the situation and interest in its follow-up |
| Resident considers nurse’s opinion |
| Resident asks for nurse’s opinion, asks for nurse’s help in decision making, involves him/her in the reflection |
| **Theme 2:** technical communication |
| Nurse asks for a decision, a prescription, a precision about a given prescription |
| Nurse calls out results of exams (blood pressure, saturation…) |
| Nurse confirms the execution of the medical prescriptions (closed loop) |
| Nurse verifies the medical prescriptions: either in an inquiring way (by asking a question on a given prescription), or in an affirmative way (by repeating a prescription aloud) (check-back) |
| Nurse plans the sequence of the actions out loud (prioritization) |
| Nurse uses the patient to communicate with the resident |
| Resident gives a medical order |
| Resident calls out results of exams (auscultation, lab, EKG,…) |
| Resident plans aloud the sequence of the actions (prioritization) |
| Resident verifies that medical orders are in progress |
| Resident uses the patient to communicate with the nurse |
| **Theme 3:** manifestations regarding team building (positive or negative) |
| Nurse provides a positive feedback to the resident (about a diagnosis, a prescription, a behaviour), valorises, supports, thanks the resident |
| Nurse provides a negative feedback to the resident (about a diagnosis, a prescription, a behaviour) |
| Nurse explains a fact to the resident (mechanisms, knowledge) |
| Nurse helps the resident or proposes to help |
| Nurse interrupts him-/herself to respond to the resident’s request |
| Nurse demonstrates negative emotions: unequivocal words or gestures |
| Resident provides a positive feedback to the nurse (about a diagnosis, a prescription, or a behaviour), valorises, supports, thanks the nurse |
| Resident provides a negative feedback to the nurse (about a diagnosis, a prescription, or a behaviour) |
| Resident explains the situation with an educational intent (mechanisms, knowledge,… beyond simple clarification) |
| Resident helps the nurse or proposes to help |
| Resident interrupts him-/herself to respond to the nurse’s request |
| Resident demonstrates negative emotions: unequivocal words or gestures |
| **Overall impression: how much present the following characteristics are** |
| Roles are traditional |
| The team works in harmony |
| The team is efficient in patient management |
| The team has a common objective |
| The resident takes leadership of patient management |
| The nurse takes leadership of patient management |
| The resident demonstrates autonomy in patient management |
| The nurse demonstrates autonomy in patient management |
| The resident demonstrates listening to nurse |
| The nurse demonstrates listening to resident |
| The team demonstrates shared decision making and common reasoning |
